# Association of MICA Alleles with Autoimmune Thyroid Disease in Korean Children

**DOI:** 10.1155/2012/235680

**Published:** 2012-11-14

**Authors:** Won Kyoung Cho, Min Ho Jung, So Hyun Park, In Cheol Baek, Hee-Baeg Choi, Tai-Gyu Kim, Byung-Kyu Suh

**Affiliations:** ^1^Department of Pediatrics, College of Medicine and Seoul St. Mary's Hospital, The Catholic University of Korea, 505 Banpo-Dong, Seocho-Gu, Seoul 137-040, Republic of Korea; ^2^Department of Microbiology, College of Medicine and Seoul St. Mary's Hospital, The Catholic University of Korea, 505 Banpo-Dong, Seocho-Gu, Seoul 137-040, Republic of Korea; ^3^Catholic Hematopoietic Stem Cell Bank, College of Medicine and Seoul St. Mary's Hospital, The Catholic University of Korea, 505 Banpo-Dong, Seocho-Gu, Seoul 137-040, Republic of Korea

## Abstract

*Background*. Major histocompatibility complex class I chain-related gene A (MICA) is a ligand for the activating NKG2D receptor expressed on natural killer (NK) cells. We aimed to assess the association of MICA polymorphism with autoimmune thyroid disease (AITD) in Korean children. *Methods*. Eighty-one patients with AITD were recruited. We analyzed MICA polymorphisms by PCR-SSP and compared the results with those of 70 healthy controls. *Results*. In AITD, the allele frequencies of MICA∗010 (OR = 2.21; 95% CI, 1.30–3.76, *P* < 0.003, *P*_*c*_ < 0.042) were higher than those of controls. Patients who did not have thyroid-associated ophthalmopathy showed higher frequencies of MICA∗010 (OR = 2.99; 95% CI, 1.47–6.08, *P* < 0.003, *P*_*c*_ < 0.042) and lower frequencies of MICA∗008 (OR = 0.08; 95% CI, 0.01–0.62, *P* < 0.001, *P*_*c*_ < 0.014) compared to those of controls. HLA-B∗46, which shows the strongest association with AITD compared with other HLA alleles, showed the strongest linkage disequilibrium with MICA∗010. Analyses of the associations between MICA∗010 and HLA-B∗46 with AITD suggest an association of the MICA allele with AITD. *Conclusions*. Our results suggest that innate immunity might contribute to the pathogenesis of AITD.

## 1. Introduction

The structure and function of endocrine glands (i.e., well-developed peripheral blood vessels and hormone secretion) make them particularly susceptible to autoimmune disease [1]. Autoimmune thyroid disease (AITD) may occur when genetically susceptible individuals are exposed to environmental modulating triggers such as infection, iodine, or stress [2].

Hashimoto disease (HD) seems to involve a CD4 Th1 response. The effects of antibodies and effector T cells specific for thyroid antigens lead to the progressive destruction of normal thyroid tissue [3]. The autoimmune response in Graves' disease (GD) is biased towards a CD4 Th2 response and is focused on antibody production [3]. The production of anti-TSH-receptor antibodies promotes chronic overproduction of thyroid hormone [3]. The fact that GD and HD are commonly observed in the same family tree reflects a similar genetic basis for the 2 diseases [4, 5].

The major histocompatibility complex (MHC) class I chain-related gene A (MICA) is located within the human MHC locus, centromeric to HLA-B and telomeric to HLA-DRB1 [6]. In response to stress (e.g., viral infection, DNA damage, or malignant transformation), MICA proteins are expressed on the surface of freshly isolated gastric epithelial and endothelial cells and fibroblasts, where they engage the activating receptor NKG2D, which is expressed by NK, NKT, CD8+ T, and *γδ* T cells, and activated macrophages. 

Although the relationships between NKG2D and MICA polymorphisms associated with autoimmune and neoplastic diseases, including ankylosing spondylitis [7], Behçet's disease [8], psoriasis vulgaris [9], and Kawasaki's disease [10], have been defined, this is not the case for the association of MICA alleles with AITD.

In the present study, we aimed to assess the association between MICA polymorphisms, as classified by the World Health Organization (WHO), with Korean children with AITD. We also evaluated the frequencies of the MICA allele according to age, sex, and thyroid-associated ophthalmopathy (TAO) in patients with AITD.

## 2. Methods

### 2.1. Subjects

The present study included 81 patients diagnosed with AITD (36 HD; 45 GD) who were treated in the pediatric endocrine clinic of Seoul St. Mary's Hospital and Yeouido St. Mary's Hospital between March 2009 and January 2012. Seventy-three patients were also included in previously by us [11]. For comparison, 70 genetically unrelated healthy Korean adults without a history of AITD were studied as a control group. The control subjects consisted mainly of staff and students from the Medical College of the Catholic University of Korea. All subjects gave informed consent for a genetic study. The institutional review board of the Catholic University of Korea approved our study.

HD was diagnosed when at least 3 of the following Fisher's criteria [12] were met: (1) goiter, (2) diffuse goiter and decreased radionuclide uptake during thyroid scan, (3) presence of either circulating thyroglobulin or microsomal autoantibodies or both, and (4) hormonal evidence of hypothyroidism. The diagnosis of GD was based on the confirmation of clinical symptoms and the biochemical confirmation of hyperthyroidism, including the diagnosis of goiter, the elevated ^131^I uptake by the thyroid gland, the presence of antibodies reactive against the TSH receptor, and the elevated thyroid hormone levels. Patients with other forms of autoimmune diseases, hematologic diseases or endocrine diseases, or both were excluded.

### 2.2. DNA Extraction

Genomic DNA was extracted from 4 mL of peripheral blood mixed with ethylenediaminetetraacetic acid (EDTA) using the AccuPrep DNA extraction Kit (Bioneer, Daejeon, Republic of Korea) and stored at −20°C.

### 2.3. PCR-SSP Analysis of MICA

PCR amplifications were performed in 10 *μ*L reaction mixtures in 96-well thin-walled trays (Nippon Genetics, Tokyo, Japan) [13]. The reaction mixtures consisted of 1.5–2.0 *μ*M sequence-specific primers, 0.1 *μ*g genomic DNA, 20 mM (NH_4_)_2_SO_4_ (Sigma, Dorset, UK), 75 mM Tris-HCl (pH 8.8) (Sigma, Dorset, UK), 200 *μ*M of each dNTP (Roche, Mannheim, Germany), 1.75 mM MgCl_2_ (Sigma, Dorset, UK), 0.22 units of Taq polymerase (Intron Biotechnology, Seongnam, Republic of Korea), and 0.15 *μ*M internal control primers that amplify an 834 bp portion of the human growth hormone gene (5′ primer, GCCTTCCCAACCATTCCCTTA; 3′ primer, GAGAAAGGCCTGGAGGATTC). 

A MyCycler Thermalcycler (Bio-Rad Inc., Hercules, CA, USA) was used to perform PCR as follows: initial denaturation at 95°C for 90 s; 10 cycles of denaturation at 95°C for 30 s, annealing at 64.5°C for 50 s, and elongation at 72°C for 20 s; 10 cycles of denaturation at 95°C for 30 s, annealing at 61.5°C for 50 s, and elongation at 72°C for 30 s; 10 cycles of denaturation at 95°C for 30 s, annealing at 60°C for 50 s, and elongation at 72°C for 40 s, followed by cooling to 20°C. Ten microliters of each reaction was electrophoresed through 1.5% agarose gels containing 2.5 *μ*g/mL ethidium bromide (Sigma Aldrich, Mo, USA) for 22 min at 250 mV in 1 × TBE buffer.

### 2.4. Clinical Characteristics of Patients and Associations with MICA Polymorphisms

We evaluated the MICA allele frequencies according to age, sex, and thyroid-associated ophthalmopathy (TAO) in AITD. The diagnosis of TAO was based on the presence of typical clinical features and was classified according to the criteria of the American Thyroid Association [14, 15]. Patients with no symptoms or with only the lid lag sign were considered to have non-TAO, whereas those with soft tissue changes, proptosis or extraocular muscle dysfunction, or both were considered to have a disease of the eye [16].

### 2.5. Statistical Analysis

The Statistical Package for Social Sciences 12.0 for Windows (SPSS, Chicago, IL, USA) was used for statistical calculations. Values are expressed as the mean ± standard deviation. The significance of the difference in the distribution of MICA alleles was assessed using the chi-square and Fisher's exact tests. Multiplication of the *P* value by the number of comparisons for calculating corrected *P* value (*P*_*c*_) was done, and *P*_*c*_ below 0.05 is considered significant. The relative risk was calculated using the Woolf formula and Handan's modification for cases in which the variables included zero. The frequencies of HLA-A, B, C, DRB1, and MICA were calculated by direct counting, and the patterns of two locus haplotypes and Hardy-Weinberg equilibrium for MICA were estimated using the maximum-likelihood method with the expectation-maximization (EM) algorithm using PYPOP [17, 18]. 

In the analyses of the association of AITD, MICA∗010, and HLA alleles, the basic data are tabulated in a 2 × 4 table. The data were analyzed using test numbers (1)–(8) (Table 4), including stratification of each of the associated factors against each other and are presented in a series of 2 × 2 tables. A stronger association of 1 allele is established when it is significantly associated with the condition of individuals positive or negative for the other associated allele. The opposite holds true for the reciprocal stratification. Correction factors are stated in [19].

## 3. Results

### 3.1. Clinical Characteristics of the Subjects

The subject group consisted of 81 patients (65 girls and 16 boys) with AITD. The mean age of the patients was 11.1 ± 2.7 years. AITD was categorized as HD (*n* = 36) and GD (*n* = 45). Twenty-three patients were diagnosed with TAO, 21 with GD (21/45, 46.6%), and 2 with HD (2/36, 5.5%). 

### 3.2. Allele Frequencies of MICA in the AITD and Control Groups

For patients with AITD, the allele frequencies of MICA*010 (OR = 2.21; 95% CI, 1.30–3.76, *P* < 0.003, *P*_*c*_ < 0.042) were higher than the control group (Table 1). 

### 3.3. Difference in MICA Allele Frequencies according to TAO

Significant differences were not detected in MICA allele frequencies according to sex and age in patients with AITD (data not presented). Among patients with GD, those without TAO (*n* = 24) exhibited higher frequencies of MICA*010 (OR = 2.99; 95% CI, 1.47–6.08, *P* < 0.003, *P*_*c*_ < 0.042) and lower frequencies of MICA*008 (OR = 0.08; 95% CI, 0.01–0.62, *P* < 0.001, *P*_*c*_ < 0.014) than did the normal control group. There were no significant differences in the frequencies of MICA allele frequencies between the non-TAO and TAO groups (Table 2) among patients with GD. 

### 3.4. MICA and HLA Alleles and Genetic Susceptibility to AITD

For patients with AITD, two-locus haplotypes with frequencies higher than 3% were as follows: MICA*010-HLA-A*02 (26.6%), MICA*010-HLA-B*46 (22.5%), MICA*010-B*62 (8.0%), MICA*010-HLA-C*01 (22.9%), MICA*010-HLA-C*04 (4.2%), MICA*010-HLA-C*09 (3.5%), MICA*010-HLA-DR*08 (16.0%), and MICA*010-HLA-DR*09 (4.9%). All of these two-locus haplotypes showed high linkage disequilibrium (Table 3), and the strongest linkage disequilibrium was observed between MICA*010 and HLA-B*46. In control subjects, the two-locus haplotypes with frequencies higher than 3% were as follows: MICA*010-HLA-A*02 (8.5%), MICA*010-HLA-B*46 (6.6%), MICA*010-B*62 (9.56%), MICA*010-HLA-C*01 (8.8%), MICA*010-HLA-C*09 (3.0%), and MICA*010-HLA-DR*08 (5.9%) (data not presented).

### 3.5. Association of MICA*010 and HLA-B*46 with AITD

We investigated the relative strength of the associations of MICA*010 and HLA-B*46 with AITD. Test numbers (1) and (2) showed that both were associated with AITD. HLA-B*46 showed an association in MICA*010-negative ones (test numbers (6)). According to test number (8), carrying both alleles gives an association with AITD (Table 4) [20]. 

## 4. Discussion

Various hypotheses have been proposed to explain the role of MICA in autoimmune diseases. With respect to tissue subjected to stress, the recognition of MICA by cells expressing NKG2D might participate in the induction of autoimmune diseases. Autoreactive immune cells expressing MICA are also recognized and killed by NKG2D-activated NK cells. If the autoreactive immune cells escape from the attack of the NKG2D-bearing cells, they have the potential to proliferate and induce pathological autoimmunity [21].

In our present study, the frequency of the MICA*010 allele in patients with AITD was higher than that in the control group. Those of MICA*004 (OR = 0.16; 95% CI, 0.04–0.76, *P* < 0.015, *P*_*c*_ < 0.21) and MICA*008 (OR = 0.42; 95% CI, 0.22–0.81, *P* < 0.010, *P*_*c*_ < 0.14) in patients with AITD were lower than in the control group but not significant (Table 1). In a recent study, the frequencies of the MICA A5 and A5.1 alleles were higher and lower, respectively, for Chinese children with GD compared with the control groups [22]. According to the nomenclature recommended by the WHO (http://www.anthonynolan.org.uk/HIG/, in July 2012), MICA*010 is included with MICA A5, and MICA*008 is included with MICA A5.1 [23]. Therefore, the results of our study are similar to those of the study conducted in China, although there are no reports on the association between MICA and AITD in other populations.

More than 50 human MICA alleles have been recognized, and the frequencies of each allele are different in different populations [23]. MICA*008 is the most common allele in Caucasians with an allele frequency of 46%, but it is very rare in Koreans with an allele frequency of 10%. In Koreans, MICA*010 is the most common allele. The MICA gene comprises 6 exons, which encode a leader peptide, 3 distinct extracellular domains (*α*1, 2, 3), a transmembrane domain, and a cytoplasmic tail, respectively. Single amino acid substitutions occur frequently within exons 2–4, which encode the 3 extracellular domains [24].

In MICA*010, a proline residue is substituted for an arginine residue at position 6 in the first *β* strand of the *α*1 domain. The single proline substitution disrupts the secondary structure of the *β* sheet, interfering with the folding of the protein, and abolishes its expression by inhibiting its ability to translocate to the cell surface [25]. Therefore, NK cell may be more downregulated in MICA*010-positive patients with AITD than in the control groups. 

MICA*008 is expressed as a soluble molecule, because it is encoded by a sequence that contains a single-nucleotide insertion, which results in the synthesis of a protein truncated within the transmembrane region. This particular form of MICA*008, which is distinct from products of other alleles of MICA, is generated by a different mechanism of release from the cell surface involving enclosure in exosomes [26, 27]. Elevated levels of soluble MICA have been detected in the sera of patients with various types of cancer, and the levels of soluble MICA can be used as a diagnostic marker for cancer progression. The release of soluble MICA from tumor cells reduces the cell surface density of MICA, leading to reduced susceptibility to NKG2D-mediated cytotoxicity and systemic downregulation of the cell surface expression of NKG2D on effector cells [27]. In our study, the frequencies of MICA*008 in patients with AITD tend to be lower than the control group. These results may suggest that NKG2D-bearing cell activation is more upregulated in patients with AITD than the control groups.

In patients with rheumatoid arthritis, large amounts of soluble MICA, presumably derived from synoviocytes, were detected in serum, and they might be stimulating autoreactive T cells. These data suggest a potential role for MICA gene polymorphisms in the susceptibility to autoimmune disease [28, 29]. Along with ethnic diversity of the distribution of MICA alleles, the allelic frequencies of MICA*019, the other soluble form of MICA, are very low in the Korean population. Therefore, no significant difference was observed in the allele frequencies of MICA*019 between the AITD and the control groups.

TAO is a common inflammatory autoimmune disease of the orbit, which is very closely associated with GD and is considered an autoimmune response against one or several ophthalmological autoantigens shared with the thyroid. TAO sometimes occurs in patients with euthyroid or hypothyroid chronic autoimmune thyroiditis [30]. Nearly 80% of GD patients exhibit symptoms involving the eyes, and half of these patients exhibit clinically significant TAO [31]. In our present study, 23 of 81 patients were diagnosed with TAO and included 21/45 (46.6%) patients with GD and 2 (2/36, 5.5%) patients with HD. Although the precise pathophysiology of TAO remains unclear, it is likely to reflect an autoimmune reaction involving sensitized T lymphocytes and autoantibodies directed against specific orbital or thyroid-and-orbital shared antigens [30].

Several studies have reported associations between TAO and MHC class I [32], II [33], and CTLA4 [34]; however, association of MICA alleles with TAO have not been reported. We investigated the frequency of MICA alleles according to the presence of TAO. We observed an increased frequency of the MICA*010 allele and a decreased allele frequency of MICA*008 in the non-TAO GD group compared with controls. Therefore, we suggest that MICA*010 and MICA*008 may be protective and deleterious alleles, respectively, in patients with TAO. 

MICA might be in linkage disequilibrium with HLA because of its proximity to those of HLA-B and HLA-C loci. Therefore, we investigated the two-locus haplotype frequencies between MICA*010 and HLA in the AITD group. The data indicate that the two-locus haplotype frequencies identified in order of highest to lowest were as follows: MICA*010-A*02, MICA*010-C*01, MICA*010-B*46, and MICA*010-DR*08 in patients with AITD. All of these two-locus haplotypes exhibited significant linkage disequilibrium, with the strongest observed between MICA*010 and HLA-B*46. The frequency of the MICA*010-HLA-B46 two-locus haplotype was estimated as 5.8% [35] and that of MICA*010-HLA-DR*08 was estimated as 5% [6] in the Korean population. Analysis of the disease associations of MICA*010 and HLA-B*46 with AITD revealed the associations of MICA*010 and HLA-B*46 with AITD. Although the MICA*010 association was weaker than that of HLA-B*46, these results may suggest an association of the MICA allele with AITD. 

In this study, we observed increased frequency of the MICA*010 in Korean pediatric patients with AITD compared with the controls. Non-TAO GD patients exhibited higher frequencies of MICA*010 and lower frequencies of MICA*008 compared with the controls. Our study has a limitation because of the small number of cases and controls. The power of our study according to PASS, 2010 was 53%. However, this information may provide basic data on the association of MICA polymorphism with pathogenesis of AITD. A larger and statistically more powerful molecular study will be required to confirm these results in a near future.

## Figures and Tables

**Table 1:** MICA allele frequencies in patients with AITD and controls.

MICA allele	Controls (2*n* = 140)	HD (2*n* = 72)	GD (2*n* = 90)	AITD (HD + GD) (2*n* = 162)
MICA*002	17 (12.1%)	7 (9.7%)	8 (8.9%)	15 (9.3%)
MICA*004	10 (7.1%)	1 (1.4%)	1 (1.1%)	2 (1.2%)
MICA*006	0 (0%)	0 (0%)	1 (1.1%)	1 (0.6%)
MICA*007	5 (3.6%)	3 (4.2%)	1 (1.1%)	4 (2.5%)
MICA*008	29 (20.7%)	8 (11.1%)	8 (8.9%)	16 (9.9%)
MICA*009	7 (5.0%)	4 (5.6%)	4 (4.4%)	8 (4.9%)
MICA*010	27 (19.3%)	23 (31.9%)	33 (36.7%)	56 (34.6%)^a^
MICA*011	4 (2.9%)	0 (0%)	0 (0%)	0 (0%)
MICA*012	14 (10.0%)	8 (11.1%)	11 (12.2%)	19 (11.7%)
MICA*017	1 (0.7%)	0 (0%)	0 (0%)	0 (0%)
MICA*019	1 (0.7%)	2 (2.8%)	3 (3.3%)	5 (3.1%)
MICA*027	14 (10.0%)	7 (9.7%)	13 (14.4%)	20 (12.3%)
MICA*045	1 (0.7%)	3 (4.2%)	0 (0%)	3 (1.9%)
MICA*049	10 (7.1%)	6 (8.3%)	7 (7.8%)	13 (8%)

MICA: major histocompatibility complex (MHC) class I chain-related gene A; AITD: autoimmune thyroid diseases; HD: Hashimoto's disease; GD: Graves' disease; C: healthy control subjects; allele frequencies of MICA were in the Hardy-Weinberg equilibrium (*P* < 0.349); ^a^ *P* < 0.003 (*P*_*c*_ < 0.042), OR = 2.21 (95% confidence interval (CI), 1.30–3.76) versus C.

**Table 2:** Allele frequencies of MICA in patients with GD with and without TAO.

MICA allele	Controls (2*n* = 140)	Non-TAO (2*n* = 48)	TAO (2*n* = 42)	GD (2*n* = 90)
MICA*002	17 (12.3%)	2 (4.2%)	6 (14.3%)	8 (9.8%)
MICA*004	10 (7.1%)	1 (2.1%)	0 (0%)	1 (1.2%)
MICA*006	0 (0%)	1 (2.1%)	0 (0%)	1 (1.2%)
MICA*007	5 (3.6%)	0 (0%)	1 (2.4%)	1 (1.2%)
MICA*008	29 (20.7%)	1 (2.1%)^a^	7 (16.7%)	8 (8.9%)
MICA*009	7 (5.0%)	3 (6.3%)	1 (2.4%)	4 (4.4%)
MICA*010	27 (19.3%)	20 (41.7%)^b^	13 (31.0%)	33 (36.7%)
MICA*011	4 (2.9%)	0 (0%)	0 (0%)	0 (0%)
MICA*012	14 (10.0%)	8 (16.7%)	3 (7.1%)	11 (12.2%)
MICA*017	1 (0.7%)	0 (0%)	0 (0%)	0 (0%)
MICA*019	1 (0.7%)	2 (4.2%)	1 (2.4%)	3 (3.3%)
MICA*027	14 (10.0%)	6 (12.5%)	7 (16.7%)	13 (14.4%)
MICA*045	1 (0.7%)	0 (0%)	0 (0%)	0 (0%)
MICA*049	10 (7.1%)	4 (8.3%)	3 (7.1%)	7 (7.8%)

MICA: major histocompatibility complex (MHC) class I chain-related gene A; TAO: with thyroid associated ophthalmopathy; non-TAO: without thyroid associated ophthalmopathy; GD: Graves' disease; C: healthy control subjects; allele frequencies of MICA were in the Hardy-Weinberg equilibrium (*P* < 0.349); ^a^ *P* < 0.001 (*P*_*c*_ < 0.014), OR = 0.08 (95% CI, 0.01–0.62) versus C; ^b^ *P* < 0.003 (*P*_*c*_ < 0.042), OR = 2.99 (95% CI, 1.47–6.09) versus C.

**Table 3:** Two-locus haplotype frequencies (>3%) of MICA and HLA genes in patients with AITD.

Haplotypes	Patients HF (%)	LD
MICA*010-A*02	26.7	0.115
MICA*010-B*46	22.5	0.138
MICA*010-B*62	8.0	0.046
MICA*010-C*01	22.9	0.096
MICA*010-C*09	4.2	0.01
MICA*010-C*04	3.5	0.018
MICA*010-DR*08	16.0	0.071
MICA*010-DR*09	4.9	0.012

MICA: major histocompatibility complex (MHC) class I chain-related gene A; HLA: human leukocyte antigen; AITD: autoimmune thyroid diseases; HF: haplotype frequencies; LD: linkage disequilibrium.

**Table:** (a) Basic data for MICA*010 and B*46

Factor A	Factor B	Number	Control (2*n*)
MICA*010	HLA-B*46	AITD (2*n*)	
+	+	22	7
+	−	34	20
−	+	16	2
−	−	90	111

		162	140

**Table:** (b) Two-by-two comparisons

Comparison	*a*	*b*	*c*	*d*	OR	*P* (*P*_*c*_)	No.	Conclusion
A versus non-A	56	106	27	113	2.211	0.003 (0.02)	(1)	MICA*010 associated
B versus non-B	38	124	9	131	4.461	0.00005 (0.0003)	(2)	B*46 associated
++ versus −+	22	16	7	2	0.393	0.449 (2.694)	(3)	MICA*010 not associated in B*46 (+)
+− versus − −	34	90	20	111	2.097	0.021 (0.126)	(4)	MICA*010 associated in B*46 (−)
++ versus +−	22	34	7	20	1.849	0.326 (1.956)	(5)	B*46 not associated in MICA*010 (+)
−+ versus − −	16	90	2	111	9.867	0.0003 (0.002)	(6)	B*46 associated in MICA*010 (−)
+− versus −+	34	16	20	2	4.706	0.043 (0.258)	(7)	MICA*010 and B*46 associations differ
++ versus − −	22	90	7	111	3.876	0.002 (0.02)	(8)	Combined MICA*010 and B*46 association

MICA: major histocompatibility complex (MHC) class I chain-related gene A; HLA: human leukocyte antigen; AITD: autoimmune thyroid diseases.
